# Supplementary material for: Uncovering bifurcation behaviors of biochemical reaction systems from network topology
Source: Sci Rep. 2025 Jul 29;15:27596. doi: 10.1038/s41598-025-10688-6 (PMC12307687; doi:10.1038/s41598-025-10688-6)
Supplement: Supplementary file 1 — Supplementary Information. [file 41598_2025_10688_MOESM1_ESM.pdf]

Supplementary Materials for "Uncovering Bifurcation Behaviors of Biochemical Reaction Systems from Network Topology"

Yong-Jin Huang[1,2*], Takashi Okada[1,3], and Atsushi Mochizuki[1,2]

[1]Institute for Life and Medical Sciences, Kyoto University, Kyoto, Japan
[2]Department of Biophysics, Graduate School of Science, Kyoto University, Kyoto, Japan
[3]Interdisciplinary Theoretical and Mathematical Sciences Program (iTHEMS), RIKEN, Wako, Japan
*Correspondence to Yong-Jin Huang; Email: yongjin.huang.83r@st.kyoto-u.ac.jp

Appendix A: Buffering structures and the localization principle

In this appendix, we are going to formally define *buffering structures* of chemical reaction networks and to explain the localization principle arising from buffering structures. The content is virtually a summary of previous studies [1, 2, 3] to make the present paper self-contained. To help readers better follow up the theory, we will first introduce (i) the theory of Structural Sensitivity Analysis, give (ii) the definition of buffering structures, and finally state and prove (iii) localization principle arising from buffering structures.

I. Structural Sensitivity Analysis

We shall first start from the ordinary differential equation

$$\dot{x} = \nu\, \boldsymbol{r}(\boldsymbol{x}; \boldsymbol{k}) =: f(\boldsymbol{x}; \boldsymbol{k}) \tag{29}$$

as given in the beginning of Method section in the main text. We may assume the parameter $\boldsymbol{k}$ in the reaction rate function $f(\boldsymbol{x}; \boldsymbol{k}) = \nu\, \boldsymbol{r}(\boldsymbol{x}; \boldsymbol{k})$ is a vector in $\mathbb{R}^N$, representing enzyme activities or any other reaction-specific factors. The specificity infers that $\frac{\partial r_n}{\partial k_{n'}} = 0$ if and only if $n = n'$.

Suppose that the equilibrium manifold is parameterized by the parameter $\boldsymbol{k}$ and the conserved quantity $\boldsymbol{\eta} := -D^{\mathsf{T}}\boldsymbol{x}$, locally near a fixed equilibrium point $\boldsymbol{x}_0 \in \mathbb{R}^M$. Besides, put C to be a full-ranked matrix such that $\langle -C \rangle = \ker \nu$. Remark that we multiply the column vectors of kernel basis matrix and cokernel basis matrix (that is, C and D) just for simplicity of the following computation. With the parametrization of equilibria, we have

$$\boldsymbol{0} \equiv \nu \boldsymbol{r}(\overline{\boldsymbol{x}}(\boldsymbol{k}, \boldsymbol{\eta}); \boldsymbol{k}), \tag{30}$$

which is followed by the existence of a mapping $\boldsymbol{\mu}(\boldsymbol{k}, \boldsymbol{\eta}) \in \mathbb{R}^{\dim \ker \nu}$ locally such that

$$-C\boldsymbol{\mu}(\boldsymbol{k}, \boldsymbol{\eta}) \equiv \boldsymbol{r}(\overline{\boldsymbol{x}}(\boldsymbol{k}, \boldsymbol{\eta}); \boldsymbol{k}). \tag{31}$$

Taking the partial derivative with respect to $\boldsymbol{k}$, one obtains

$$-C\frac{\partial \boldsymbol{\mu}}{\partial \boldsymbol{k}} = \frac{\partial \boldsymbol{r}}{\partial \boldsymbol{x}}\frac{\partial \overline{\boldsymbol{x}}}{\partial \boldsymbol{k}} + \frac{\partial \boldsymbol{r}}{\partial \boldsymbol{k}}. \tag{32}$$

Notice that due to the (enzyme) specificity, the matrix $\frac{\partial \boldsymbol{r}}{\partial \boldsymbol{k}}$ is diagonal; moreover, we may assume that $\frac{\partial \boldsymbol{r}}{\partial \boldsymbol{k}} = I$ at $\boldsymbol{x}_0$, since the unit are flexible to re-scale locally. As such, the equation is rewritten to be

$$-I = \frac{\partial \boldsymbol{r}}{\partial \boldsymbol{x}} \frac{\partial \overline{\boldsymbol{x}}}{\partial \boldsymbol{k}} + C \frac{\partial \boldsymbol{\mu}}{\partial \boldsymbol{k}}. \tag{33}$$

Besides, with $\boldsymbol{\eta} \equiv -D^{\mathsf{T}}\, \overline{\boldsymbol{x}}(\boldsymbol{k}, \boldsymbol{\eta})$ by definition, we have

$$-I = \frac{\partial}{\partial \boldsymbol{\eta}} \left(D^{\mathsf{T}}\, \overline{\boldsymbol{x}}(\boldsymbol{k}, \boldsymbol{\eta}) \right) = D^{\mathsf{T}} \frac{\partial \overline{\boldsymbol{x}}}{\partial \boldsymbol{\eta}} \tag{34}$$

and

$$O = \frac{\partial}{\partial \boldsymbol{k}} \left(D^{\mathsf{T}}\, \overline{\boldsymbol{x}}(\boldsymbol{k}, \boldsymbol{\eta}) \right) = D^{\mathsf{T}} \frac{\partial \overline{\boldsymbol{x}}}{\partial \boldsymbol{k}}, \tag{35}$$

where O denotes a zero matrix. Taken the partial derivatives by $\boldsymbol{\eta}$, Eq. (31) implies

$$-C \frac{\partial \boldsymbol{\mu}}{\partial \boldsymbol{\eta}} = \frac{\partial \boldsymbol{r}}{\partial \boldsymbol{x}} \frac{\partial \overline{\boldsymbol{x}}}{\partial \boldsymbol{\eta}} = -\frac{\partial \boldsymbol{r}}{\partial \boldsymbol{x}} D^{\mathsf{T}}. \tag{36}$$

Combine equations (33), (34), (35), and (36), we have

$$-I = \underbrace{\left(\begin{array}{c|c} \frac{\partial \boldsymbol{r}}{\partial \boldsymbol{x}} & C \\ \hline D^{\mathsf{T}} & \end{array} \right)}_{=\boldsymbol{A}} \left(\begin{array}{c|c} \frac{\partial \overline{\boldsymbol{x}}}{\partial \boldsymbol{k}} & \frac{\partial \overline{\boldsymbol{x}}}{\partial \boldsymbol{\eta}} \\ \hline \frac{\partial \boldsymbol{\mu}}{\partial \boldsymbol{k}} & \frac{\partial \boldsymbol{\mu}}{\partial \boldsymbol{\eta}} \end{array} \right). \tag{37}$$

Then, when the matrix $\boldsymbol{A}$ is invertible at $\boldsymbol{x}_0$, the equation (37) implies that the system response $(\delta \boldsymbol{x}, \delta \boldsymbol{\mu})$ to infinitesimal perturbation $(\delta \boldsymbol{k}, \delta \boldsymbol{\eta})$ is given by

$$\left(\begin{array}{c} \delta \boldsymbol{x} \\ \hline \delta \boldsymbol{\mu} \end{array} \right) = \left(\begin{array}{c|c} \frac{\partial \overline{\boldsymbol{x}}}{\partial \boldsymbol{k}} & \frac{\partial \overline{\boldsymbol{x}}}{\partial \boldsymbol{\eta}} \\ \hline \frac{\partial \boldsymbol{\mu}}{\partial \boldsymbol{k}} & \frac{\partial \boldsymbol{\mu}}{\partial \boldsymbol{\eta}} \end{array} \right) \left(\begin{array}{c} \delta \boldsymbol{k} \\ \hline \delta \boldsymbol{\eta} \end{array} \right) = -\boldsymbol{A}^{-1} \left(\begin{array}{c} \delta \boldsymbol{k} \\ \hline \delta \boldsymbol{\eta} \end{array} \right). \tag{38}$$

We remark that $\delta \boldsymbol{x}$ and $\delta \boldsymbol{\mu}$ respectively represent the changes in the chemical concentrations and the reaction rates (since $\boldsymbol{r} = -C\boldsymbol{\mu}$).

II. Buffering structures in chemical reaction network

Next, we are going to provide two ways of defining buffering structures in a chemical reaction network. The two definitions are equivalent when the chemical reaction network is *regular*, that is, $\det \boldsymbol{A} \not\equiv 0$. It is reasonable to consider regular networks only; after all, when a network is not regular, then the system is unstable everywhere in the state space (by **Theorem 1**), which is unrealistic in cell biology.

The first definition is based on an index that depends on the network topology, which highly facilitates the identification of buffering structures when it is done manually. The second definition is based on the matrix representation of $\boldsymbol{A}$. It comes in handy in mathematical derivations as well as algorithms that identify buffering structures, whereas it requires the kernel basis and cokernel basis (that is, matrices C and D in Eq. (15)) to be properly chosen.

Adopting the settings in the Method section, we suppose that the dynamics of a chemical reaction network follows equation (13). Let $\gamma = X_\gamma \cup R_\gamma$ be a subnetwork of the considered network, where X_γ and R_γ respectively denote the collections of chemicals and reactions in the subnetwork γ. Let

$\boldsymbol{x}_1$ and $\boldsymbol{x}_2$ be vectors which denote the concentrations of chemicals in and out of X_γ, respectively, and without loss of generality we may assume the chemicals to be ordered such that $\boldsymbol{x} = (\boldsymbol{x}_1, \boldsymbol{x}_2)$. Similarly, let $\boldsymbol{r}_1(\boldsymbol{x})$ and $\boldsymbol{r}_2(\boldsymbol{x})$ be the vectors of reaction rates in and out of R_γ. Then, the dynamics as described by Eq. (13) is rewritten into

$$\begin{pmatrix} \dot{\boldsymbol{x}}_1 \\ \dot{\boldsymbol{x}}_2 \end{pmatrix} = \underbrace{\begin{pmatrix} \nu_{11} & \nu_{12} \\ \nu_{21} & \nu_{22} \end{pmatrix}}_{=\nu} \begin{pmatrix} \boldsymbol{r}_1(\boldsymbol{x}) \\ \boldsymbol{r}_2(\boldsymbol{x}) \end{pmatrix}. \tag{39}$$

Furthermore, we put

$$(\ker \nu)_{\mathrm{supp}(\gamma)} \coloneqq \ker \nu_{11} \cap \ker \nu_{21} \tag{40}$$

and

$$P^0_\gamma(\operatorname{coker} \nu) \coloneqq \left\{ \boldsymbol{d} \;\middle|\; \begin{pmatrix} \boldsymbol{d} \\ \boldsymbol{d}' \end{pmatrix} \in \ker \nu^\mathsf{T} \text{ for some } \boldsymbol{d}' \right\}. \tag{41}$$

Then, $|(\ker \nu)_{\mathrm{supp}(\gamma)}|$ is the number of closed paths within the subnetwork γ, and $|P^0_\gamma(\operatorname{coker} \nu)|$ is the number of conserved quantities involved with the subnetwork γ. We then define an index by

$$\begin{aligned} \chi(\gamma) &\coloneqq |R_\gamma| - |X_\gamma| + |P^0_\gamma(\operatorname{coker} \nu)| - |(\ker \nu)_{\mathrm{supp}(\gamma)}| \\ &= |(\text{reactions in } \gamma)| - |(\text{chemicals in } \gamma)| \\ &\qquad + |(\text{conserved quantities in } \gamma)| - |(\text{loops in } \gamma)|, \end{aligned} \tag{42}$$

where $|S|$ denotes the dimension when S is a linear space, and it denotes the cardinal number when S is a finite set here. With the index, have the definition

Definition 1 *A subnetwork γ is a buffering structure if it satisfies the following two properties:*

1. *(output-completeness) All the reactions regulated by any chemicals of γ are included in γ; namely, $\frac{\partial \boldsymbol{r}_2}{\partial \boldsymbol{x}_1} \equiv 0$.*
2. *(zero index) The subnetwork satisfies $\chi(\gamma) = 0$.*

or, equivalently,

Definition 2 *A subnetwork γ is a buffering structure if there exist full-ranked matrices C and D with $\langle C \rangle = \ker \nu$ and $\langle D \rangle = \ker \nu^\mathsf{T}$ and permutation matrices P_r, P_c such that*

$$P_r \boldsymbol{A} P_c = \left(\begin{array}{c|c} \boldsymbol{A}_\gamma & * \\ \hline & \boldsymbol{A}_{\gamma^c} \end{array} \right),$$

where $\boldsymbol{A}_\gamma$ is a square block and γ^c is the complement of γ.

To see the equivalence, we may let γ be an arbitrarily given subnetwork, and let C_{11} and D_{11} be two matrices with independent column vectors such that $\langle C_{11} \rangle = (\ker \nu)_{\mathrm{supp}(\gamma)}$ and $\langle D_{11} \rangle = P^0_\gamma(\operatorname{coker} \nu)$. Then, there exist a kernel basis C and a cokernel basis D in the forms

$$C = \begin{pmatrix} C_{11} & C_{12} \\ & C_{22} \end{pmatrix}, \qquad D = \begin{pmatrix} D_{11} & \\ D_{12} & D_{22} \end{pmatrix}. \tag{43}$$

With row- and column-rearrangement, we have the matrix $\boldsymbol{A}$ in the form

$$P_r \boldsymbol{A} P_c = \left(\begin{array}{c|c|c|c} \frac{\partial \boldsymbol{r}_1}{\partial \boldsymbol{x}_1} & C_{11} & \frac{\partial \boldsymbol{r}_1}{\partial \boldsymbol{x}_2} & C_{12} \\ \hline {D_{11}}^\intercal & & {D_{21}}^\intercal & \\ \hline \frac{\partial \boldsymbol{r}_2}{\partial \boldsymbol{x}_1} & & \frac{\partial \boldsymbol{r}_2}{\partial \boldsymbol{x}_2} & C_{22} \\ \hline & & {D_{22}}^\intercal & \end{array}\right). \tag{44}$$

Put

$$\boldsymbol{A}_\gamma = \begin{array}{c} \overbrace{\hphantom{\frac{\partial \boldsymbol{r}_1}{\partial \boldsymbol{x}_1}}}^{|X_\gamma|} \quad \overbrace{\hphantom{C_{11}}}^{|(\ker \nu)_{\mathrm{supp}(\gamma)}|} \\ \left(\begin{array}{c|c} \frac{\partial \boldsymbol{r}_1}{\partial \boldsymbol{x}_1} & C_{11} \\ \hline {D_{11}}^\intercal & \end{array}\right) \begin{array}{l} \} \, |R_\gamma| \\ \} \, |P^0_\gamma(\operatorname{coker} \nu)| \end{array} \end{array}. \tag{45}$$

With Eq. (44), we see that γ is output-complete if and only if

$$P_r \boldsymbol{A} P_c = \left(\begin{array}{c|c} \boldsymbol{A}_\gamma & * \\ \hline & \boldsymbol{A}_{\gamma^c} \end{array}\right), \tag{46}$$

and Eq. (45) shows that $\boldsymbol{A}_\gamma$ is a square block if and only if $\chi(\gamma) = 0$. This shows the equivalence.

Recently, some packages have been released for the identification of buffering structures on Python [4]. The identification of buffering structures is hence feasible even for large networks in pathway databases, in which a pathway map can include hundreds of metabolites [5].

III. Localization principle arising from buffering structures

Buffering structures are known for confining system responses to parameter changes within itself in two senses. When the system is not at a bifurcation point, the equilibrium dynamics follows the *law of localization* [6, 7]—parameter changes in a buffering structure do not affect the chemicals outside the buffering structure. When the system bifurcates in response to some bifurcation parameter, then we have the *localization principle for bifurcation* [2, 3], stating that the equilibrium manifold bifurcates only in the chemicals (axes) of the buffering structure γ only when the corresponding square block $\boldsymbol{A}_\gamma$ becomes singular. In this subsection, we clarify the two theorems. The proofs are adopted from the references above but condensed and with notations in the present papers just for readers.

A. Law of localization in structural sensitivity analysis

When the matrix $\boldsymbol{A}$ is invertible at $\boldsymbol{x}_0$, the Eq. (37) implies that the system response $(\delta\boldsymbol{x}, \delta\boldsymbol{\mu})$ to infinitesimal perturbation $(\delta\boldsymbol{k}, \delta\boldsymbol{\eta})$ is given by

$$\left(\begin{array}{c} \delta\boldsymbol{x} \\ \hline \delta\boldsymbol{\mu} \end{array}\right) = \left(\begin{array}{c|c} \frac{\partial \overline{\boldsymbol{x}}}{\partial \boldsymbol{k}} & \frac{\partial \overline{\boldsymbol{x}}}{\partial \boldsymbol{\eta}} \\ \hline \frac{\partial \boldsymbol{\mu}}{\partial \boldsymbol{k}} & \frac{\partial \boldsymbol{\mu}}{\partial \boldsymbol{\eta}} \end{array}\right) \left(\begin{array}{c} \delta\boldsymbol{k} \\ \hline \delta\boldsymbol{\eta} \end{array}\right) = -A^{-1} \left(\begin{array}{c} \delta\boldsymbol{k} \\ \hline \delta\boldsymbol{\eta} \end{array}\right). \tag{47}$$

We kindly remark that $\delta\boldsymbol{x}$ and $\delta\boldsymbol{\mu}$ respectively represent the changes in the chemical concentrations and the reaction rates (since $\boldsymbol{r} = -C\boldsymbol{\mu}$). Now, suppose that there is a buffering structure γ, then we have matrices P_r and P_c such that

$$P_r \boldsymbol{A} P_c = \left(\begin{array}{c|c} \boldsymbol{A}_\gamma & * \\ \hline & \boldsymbol{A}_{\gamma^c} \end{array}\right) \tag{48}$$

with $\boldsymbol{A}_\gamma \in \mathbb{R}^{W\times W}$, where $W = |R_\gamma| + |P^0_\gamma(\operatorname{coker}\nu)| = |X_\gamma| + |(\ker\nu)_{\operatorname{supp}(\gamma)}|$. The constructions of the permutation matrices can be given according to the next paragraph.

Adopting the settings of equation (39), we let $\boldsymbol{x} = (\boldsymbol{x}_1, \boldsymbol{x}_2)$ denote the concentration of chemicals in and out of a considered buffering structure γ. The parameters can be classified as $\boldsymbol{k} = (\boldsymbol{k}_1, \boldsymbol{k}_2)$ according to whether the corresponding reactions are in the buffering structure or not. Also, we can put $\boldsymbol{\eta} = (\boldsymbol{\eta}_1, \boldsymbol{\eta}_2)$ such that $\left(\frac{\partial \boldsymbol{\eta_1}}{\partial \boldsymbol{x}_1}\right)^\mathsf{T}$ is full-ranked and $\left(\frac{\partial \boldsymbol{\eta_1}}{\partial \boldsymbol{x}_2}\right)$ is a zero matrix. Finally, we have $\boldsymbol{\mu} = (\boldsymbol{\mu}_1, \boldsymbol{\mu}_2)$ such that $\frac{\partial \boldsymbol{r}_1}{\partial \boldsymbol{\mu}_1}$ is full-ranked and $\frac{\partial \boldsymbol{r}_1}{\partial \boldsymbol{\mu}_2} = O$. Then, the matrices P_r and P_c in equation (48) can be constructed according to the permutations below:

$$P_c\,(\boldsymbol{x}, \boldsymbol{\mu})^\mathsf{T} = (\boldsymbol{x}_1, \boldsymbol{\mu}_1, \boldsymbol{x}_2, \boldsymbol{\mu}_2)^\mathsf{T} \tag{49}$$

$$P_r\,(\boldsymbol{k}, \boldsymbol{\eta})^\mathsf{T} = (\boldsymbol{k}_1, \boldsymbol{\eta}_1, \boldsymbol{k}_2, \boldsymbol{\eta}_2)^\mathsf{T} \tag{50}$$

By equation (48) and the assumption that $\boldsymbol{A}$ is invertible at $\boldsymbol{x}_0$, it is seen that

$$P_c(-A^{-1})P_r = \left(\begin{array}{c|c} -\boldsymbol{A}_\gamma{}^{-1} & * \\ \hline & -\boldsymbol{A}_{\gamma^c}{}^{-1} \end{array}\right). \tag{51}$$

Combine it with equations (37), (49) and (50), then it is shown that

$$\left(\begin{array}{c} \delta\boldsymbol{x}_1 \\ \delta\boldsymbol{\mu}_1 \\ \hline \delta\boldsymbol{x}_2 \\ \delta\boldsymbol{\mu}_2 \end{array}\right) = \left(\begin{array}{c|c} -\boldsymbol{A}_\gamma{}^{-1} & * \\ \hline & -\boldsymbol{A}_{\gamma^c}{}^{-1} \end{array}\right) \left(\begin{array}{c} \delta\boldsymbol{k}_1 \\ \delta\boldsymbol{\eta}_1 \\ \hline \delta\boldsymbol{k}_2 \\ \delta\boldsymbol{\eta}_2 \end{array}\right) \tag{52}$$

$$\Longrightarrow \left(\begin{array}{c} \delta\boldsymbol{x}_1 \\ \delta\boldsymbol{\mu}_1 \\ \hline \boldsymbol{0} \end{array}\right) = \left(\begin{array}{c|c} -\boldsymbol{A}_\gamma{}^{-1} & * \\ \hline & -\boldsymbol{A}_{\gamma^c}{}^{-1} \end{array}\right) \left(\begin{array}{c} \delta\boldsymbol{k}_1 \\ \delta\boldsymbol{\eta}_1 \\ \hline \boldsymbol{0} \end{array}\right). \tag{53}$$

Thus, when the perturbations are only given to parameters and/or conserved quantities of the buffering structure (i.e., $\delta\boldsymbol{k}_2 = 0$ and $\delta\boldsymbol{\eta}_2 = 0$), it does not exert an influence to the outside of the buffering structure (i.e., $\delta\boldsymbol{x}_2 = 0$ and $\delta\boldsymbol{\mu}_2 = 0$). This proves the law of localization arising from buffering structures in sensitivity analysis. On the other hand, when the perturbation is given to the complement of the buffering structure (namely, either of $\delta\boldsymbol{k}_2$ and $\delta\boldsymbol{\eta}_2$ is non-trivial), it in general influences the buffering structure (that is, $\delta\boldsymbol{x}_1$ and $\delta\boldsymbol{\mu}_1$).

B. Localization principle for bifurcation

We shall first stress that we focus on bifurcations of equilibria, such as transcritical, saddle-node, and pitchfork bifurcations. Bifurcations such as Hopf bifurcation are not considered in the following argument since the interest of the study is the equilibrium dynamics. Therefore, in the neighborhood of a bifurcation point $\boldsymbol{x}_0 \in \mathbb{R}^M$, we may assume that there is a piecewise $\mathcal{C}^2$ bifurcation curve

$$\zeta : \begin{array}{ccc} (-\varepsilon, \varepsilon) & \xrightarrow{\text{piecewise } \mathcal{C}^2} & \mathbb{R}^M \times \mathbb{R}^N \times \mathbb{R}^L \\ t & \longmapsto & (\boldsymbol{x}(t), \boldsymbol{k}(t), \boldsymbol{\eta}(t)) \end{array} \tag{54}$$

such that $\zeta(0) = (\boldsymbol{x}_0, \boldsymbol{k}_0, \boldsymbol{\eta}_0)$ and that $\mathcal{C}^2$ and that $\boldsymbol{A}|_{\boldsymbol{x}(t)}$ is invertible for all $t \in (-\varepsilon, \varepsilon)\backslash\{0\}$.

Once again, we set $\boldsymbol{x}(t) = (\boldsymbol{x}_1(t), \boldsymbol{x}_2(t))$, $\boldsymbol{k}(t) = (\boldsymbol{k}_1(t), \boldsymbol{k}_2(t))$, and $\boldsymbol{\eta}(t) = (\boldsymbol{\eta}_1(t), \boldsymbol{\eta}_2(t))$ accordingly with respect to a considered buffering structure. Suppose that the changes in the parameters and conserved quantities are within the buffering structure, that is, $\boldsymbol{k}_2(t)$ and $\boldsymbol{\eta}_2(t)$ are constant. Our goal is to show that $\boldsymbol{x}_2(t)$ is a constant as well.

Since $\boldsymbol{A}|_{\boldsymbol{x}(t)}$ is invertible for all $t \in (-\varepsilon, \varepsilon)\backslash\{0\}$, we see that the law of localization holds over $\zeta((-\varepsilon, \varepsilon)\backslash\{0\})$; morerover, by the continuity of the bifurcation curve, we see that $\boldsymbol{x}_2(t)$ is constant. In summary, when parameter changes and conserved quantity changes are made within a buffering structure, then the bifurcation behaviors are limited within the buffering structure.

Appendix B: the models used for method demonstration

I. A minimal example showing different types of bifurcation

For the chemical reaction network given in the Result section in the main text, we consider three different settings for the kinetics:
Model (a):

$$\begin{cases} r_1 &= k_1 \\ r_2(x_A, x_B) &= k_2\, x_A \cdot \left(\dfrac{e^{d(x_B-1)}}{1+e^{d(x_B-1)}} \right) \\ r_3(x_B) &= k_3\, x_B \\ r_4(x_A) &= k_4\, {x_A}^3 \end{cases} \tag{55}$$

Model (b):

$$\begin{cases} r_1 &= k_1 \\ r_2(x_A, x_B) &= k_2\, x_A \cdot \left(\dfrac{{x_B}^d}{1+{x_B}^d} \right) \\ r_3(x_B) &= k_3\, x_B \\ r_4(x_A) &= k_4\, {x_A}^3 \end{cases} \tag{56}$$

Model (c):

$$\begin{cases} r_1 &= k_1 \\ r_2(x_A, x_B) &= k_2\, x_A \cdot \left(\dfrac{e^{d(x_B-1)}}{1+e^{d(x_B-1)}} \right) \\ r_3(x_B) &= k_3\, x_B \\ r_4(x_A) &= k_4\, {x_A}^4 \end{cases} \tag{57}$$

In our numerical simulations, we set $k_1 = 8$, $k_2 = k_3 = k_4 = 1$. After searching for bifurcations point for $d \in [1, 5]$, we plot the bifurcation diagrams while centering the bifurcation threshold (Fig. 2).

The difference in Model (a) and Model (b) is the sigmoid function that characterizes the regulation of R_2 by X_2, which is the feedback effect of X_2 to itself. On the other hand, Model (a) and Model (c) differs in the form of the rate function r_4. Both in Model (a) and Model (c), r_4 takes a form of the mass-action kinetics but with different powers for the variable x_A.

II. A mathematical model responsible for macrophage polarizarization

In the main text, we employed our method to a network involving parts of JAK-STAT and KFκB signalings. With notations in the main text, for simplicity, we denote the system variables by $\boldsymbol{x} = (S_1, S_1^p, S_3, S_3^p, S_6, S_6^p, N, N^p)$. Then, according to the network map, the system follows

$$\frac{d}{dt}\begin{pmatrix} x_1 \\ x_2 \\ x_3 \\ x_4 \\ x_5 \\ x_6 \\ x_7 \\ x_8 \end{pmatrix} = \begin{pmatrix} -1 & 1 & & & & & & \\ 1 & -1 & & & & & & \\ & & -1 & 1 & & & & \\ & & 1 & -1 & & & & \\ & & & & -1 & 1 & & \\ & & & & 1 & -1 & & \\ & & & & & & -1 & 1 \\ & & & & & & 1 & -1 \end{pmatrix} \begin{pmatrix} r_1(x_1, x_4, x_6) \\ r_2(x_2) \\ r_3(x_2, x_3, x_4, x_8) \\ r_4(x_4) \\ r_5(x_2, x_5) \\ r_6(x_6) \\ r_7(x_4, x_7, x_8) \\ r_8(x_8) \end{pmatrix}$$

For r_n with n being an even number, the reaction is a deactivation, and we may simply put the reaction rate function as

$$r_n = k_n \cdot x_n$$

On the other hand, the activation as well as the negative regulation involve phosphorylation, which is commonly modeled as a sigmoid function [8]. Therefore, for $n = 1, 3, 5, 7$, we put

$$r_n = k_n \cdot \frac{{x_n}^{w_n}}{q_n + {x_n}^{w_n}} \cdot \left(1 - \sum_{i=1}^{8} \beta_{ni}\, \sigma(x_i)\right)$$

with $\sigma(x) = \frac{1}{1+e^{-h\cdot(x-\theta)}}$, where β_{ni}'s are non-negative numbers such that $\sum_i \beta_{ni} \geq 1$. The value of β_{ni} indicates the strength of regulating effect on R_n by X_i; in other words, $\beta_{ni} > 0$ if and only if X_i negatively regulate the activation R_n of X_n ($n = 1, 3, 5, 7$, while $i = 1, 2, \ldots, 8$). In the main text, we consider three different scenarios for the networks: (a) the wild type, (b) with SOCS3 deletion, and (c) with SOCS3 and KLF4 deletion (Fig. S1), and we simulate the three types by adjusting the values of β_{ni}'s (Table S1).

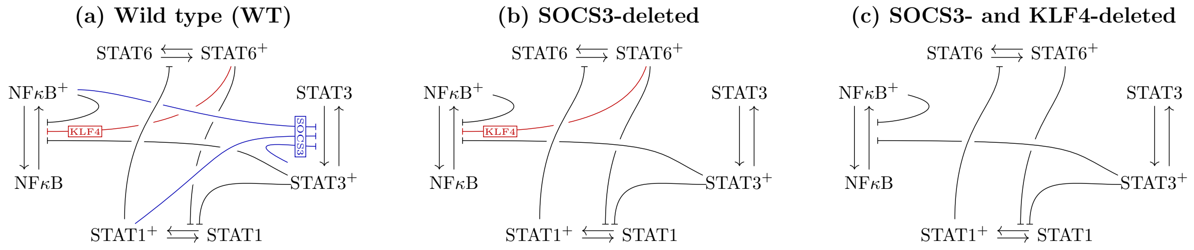

Figure S1: The reaction networks for (a) the wild type (WT), (b) the SOCS3-deleted (altered) mutants, and (c) SOCS3- and KLF4-deleted (doubly-altered) mutants.

Parameters		
$k_1 = k_2 = k_3 = k_4 = k_5 = k_6 = k_7 = k_8 = 1$, $q_1 = q_3 = q_5 = q_7 = 1$, $(w_1, w_3, w_5, w_7) = (2, 2, 2, 1)$,	$h = 5$, $\theta = 0.15$,	
(a) Wild type	(b) SOCS3-deleted	(c) SOCS3- and KLF4-deleted
$\beta = \begin{pmatrix} 0 & 0 & 0 & 0.45 & 0 & 0.45 & 0 & 0 \\ 0 & 0 & 0 & 0 & 0 & 0 & 0 & 0 \\ 0 & 0.45 & 0 & 0.045 & 0 & 0 & 0 & 0.405 \\ 0 & 0 & 0 & 0 & 0 & 0 & 0 & 0 \\ 0 & 0.9 & 0 & 0 & 0 & 0 & 0 & 0 \\ 0 & 0 & 0 & 0 & 0 & 0 & 0 & 0 \\ 0 & 0 & 0 & 0.405 & 0 & 0.405 & 0 & 0.09 \\ 0 & 0 & 0 & 0 & 0 & 0 & 0 & 0 \end{pmatrix}$	$\beta = \begin{pmatrix} 0 & 0 & 0 & 0.45 & 0 & 0.45 & 0 & 0 \\ 0 & 0 & 0 & 0 & 0 & 0 & 0 & 0 \\ 0 & 0 & 0 & 0 & 0 & 0 & 0 & 0 \\ 0 & 0 & 0 & 0 & 0 & 0 & 0 & 0 \\ 0 & 0.9 & 0 & 0 & 0 & 0 & 0 & 0 \\ 0 & 0 & 0 & 0 & 0 & 0 & 0 & 0 \\ 0 & 0 & 0 & 0.405 & 0 & 0.405 & 0 & 0.09 \\ 0 & 0 & 0 & 0 & 0 & 0 & 0 & 0 \end{pmatrix}$	$\beta = \begin{pmatrix} 0 & 0 & 0 & 0.45 & 0 & 0.45 & 0 & 0 \\ 0 & 0 & 0 & 0 & 0 & 0 & 0 & 0 \\ 0 & 0 & 0 & 0 & 0 & 0 & 0 & 0 \\ 0 & 0 & 0 & 0 & 0 & 0 & 0 & 0 \\ 0 & 0.9 & 0 & 0 & 0 & 0 & 0 & 0 \\ 0 & 0 & 0 & 0 & 0 & 0 & 0 & 0 \\ 0 & 0 & 0 & 0.405 & 0 & 0 & 0 & 0.09 \\ 0 & 0 & 0 & 0 & 0 & 0 & 0 & 0 \end{pmatrix}$

Table S1: Parameter settings for the numerical experiments of networks responsible for macrophage polarization.

Appendix C: The theorems and the proofs

The notations are adopted from the main text.

Theorem 0 *Given a kernel basis C and a cokernel basis D of the mapping ν, then there exists an orthogonal basis $\widetilde{T} \in \mathbb{R}^{(M+Q)\times(M+Q)}$ such that*

$$\widetilde{T}^{-1}\Lambda \boldsymbol{A}\widetilde{T} = \left(\begin{array}{cc|c} J_g & * & \\ \hline & D^\intercal D & \\ \hline \multicolumn{2}{c|}{*} & C^\intercal C \end{array}\right), \tag{58}$$

and hence $\det \boldsymbol{A} = \dfrac{\det(D^\intercal D)\cdot \det(C^\intercal C)}{\det \Lambda}\cdot \det J_g$.
In particular, when $\dim \operatorname{coker}\nu = 0$, *we have* $\det \boldsymbol{A} = \dfrac{\det(C^\intercal C)}{\det\Lambda}\cdot \det J_f$.

(proof) With the fact $\nu C = \mathbf{0}$ following $\langle C\rangle = \ker \nu$, it is straightforward to see that

$$\Lambda\,\boldsymbol{A} = \left(\begin{array}{c|c} \nu & D \\ \hline C^\intercal & \end{array}\right)\left(\begin{array}{c|c} \frac{\partial \boldsymbol{r}}{\partial \boldsymbol{x}} & C \\ \hline D^\intercal & \end{array}\right) = \left(\begin{array}{c|c} J_f + D^\intercal D & \\ \hline C^\intercal \frac{\partial \boldsymbol{r}}{\partial \boldsymbol{x}} & C^\intercal C \end{array}\right). \tag{59}$$

Since $D^\intercal V = 0$, $D^\intercal J_f = 0$, and $D^\intercal (D^\dagger)^\intercal = I$, we have

$$\underbrace{\left(\begin{array}{c} - \;\; V^\dagger \;\; - \\ \hline - \;\; D^\intercal \;\; - \end{array}\right)}_{=T}\left(\begin{array}{c} J_f + DD^\intercal \end{array}\right)\underbrace{\left(\begin{array}{c|c} | & | \\ V & (D^\dagger)^\intercal \\ | & | \end{array}\right)}_{=T^{-1}} = \left(\begin{array}{c|c} V^\dagger J_f V & V^\dagger J_f (D^\dagger)^\intercal \\ \hline & D^\intercal D \end{array}\right), \tag{60}$$

where T is the matrix representation of the mapping as given in (18). Put

$$\widetilde{T} := \left(\begin{array}{c|c} T & \\ \hline & I_L \end{array}\right) \tag{61}$$

in which I_L is the $L \times L$ identity matrix with $L = \dim \operatorname{coker}\nu$. Then, together with Eq. (59) and Eq. (60) as well as the definition $J_g = V^\dagger J_f V$, we obtain

$$\widetilde{T}^\intercal \Lambda \boldsymbol{A}\widetilde{T} = \left(\begin{array}{cc|c} J_g & * & \\ \hline & D^\intercal D & \\ \hline \multicolumn{2}{c|}{*} & C^\intercal C \end{array}\right) \tag{62}$$

as desired. In the special case that $\dim \operatorname{coker}\nu = 0$ (i.e., no conserved quantities), it is straightforward to see

$$\Lambda \boldsymbol{A} = \left(\begin{array}{c|c} J_f & \\ \hline * & C^\intercal C \end{array}\right), \tag{63}$$

and then it is obvious that $\det\Lambda \cdot \det \boldsymbol{A} = \det(C^\intercal C)\cdot \det J_f$. ∎

Corollary 0 *For any given state $\boldsymbol{x} \in \mathbb{R}^M$, we have*

$$\operatorname{sign}(\det\Lambda \cdot \det \boldsymbol{A}) \neq (-1)^{M-L} \implies \boldsymbol{x} \textit{ is not a stable equilibrium point.} \tag{64}$$

(proof) If $\boldsymbol{x}$ is a stable equilibrium point, then J_g is a real matrix such that each of $(M - L)$ eigenvalues possesses a negative real part. With the complex conjugate root theorem and **Theorem 1**, one obtains

$$(-1)^{(M-L)} = \mathrm{sign}(\det J_g) = \mathrm{sign}(\det \Lambda \cdot \det \boldsymbol{A}), \tag{65}$$

since the determinants of $(C^{\intercal}C)$ and $(D^{\intercal}D)$ must be positive. Hence, if the equation does not hold, then $\boldsymbol{x}$ is not a stable equilibrium point. ∎

Appendix D: Code for numerical demonstrations

Filename: symssa.py

```
import numpy as np
import sympy as sp
from string import ascii_uppercase

class CRN(object):

    """
    An object that defines a Chemical Reaction Network with a given stoichiometric matrix.
        stoi        (2D numpy.ndarray)
                    the stoichiometrix matrix with its entries assuming to be integers.
        varinames   (1D iterable)
                    the names/notations of the chemical species.
        kineticmask (string, or 2D array-like)
                    the condition in which the partial derivatives can be omitted.
    Example:
    >>> nu
    array([[ 1, -1,  1, -1],
           [ 0,  1, -1,  0],
           [ 0,  1, -1,  0]])
    >>> crn = CRN(nu)
    >>> crn.A
    Matrix([
    [         0,          0,          0,  0, -1],
    [r_{2x_{1}},          0,          0, -1,  0],
    [         0, r_{3x_{2}}, r_{3x_{3}}, -1,  0],
    [r_{4x_{1}},          0,          0,  0, -1],
    [         0,         -1,          1,  0,  0]])
    """

    def __init__(self, stoi, varinames = None, kineticmask = ">="):

        assert stoi.dtype == np.dtype('int64')
        self._nu = stoi
        self._M, self._N = stoi.shape

        if varinames is None:
            self._X = [sp.symbols('x_{%d}'%(i+1)) for i in range(stoi.shape[0])]
        else:
            self._X = [sp.symbols(sp.latex('{}'.format(var))) for var in varinames]

        if type(kineticmask) == np.ndarray:
            self._kineticmask = kineticmask.astype(bool)
        else:
            assert isinstance(kineticmask, str)
            self._kineticmask = np.array([
                [eval("{} {} 0".format(stoi[m, n], kineticmask)) for n in range(self._N)]
            for m in range(self._M)])

        self._A = _AugMat(stoi, self._X, self._kineticmask)

    @property
    def varinames(self):
        return self._X

    @varinames.setter
    def varinames(self, varinames):
        self._X = varinames
        self._A = _AugMat(self._nu, varinames, self._kineticmask)

    @property
    def kineticmask(self):
        return self._kineticmask

    @kineticmask.setter
    def kineticmask(self, kineticmask):
        self._kineticmask = kineticmask
        self._A = _AugMat(self._nu, self._X, self._kineticmask)

    @property
    def A(self):
        return self._A

    def Jacobian(self):
        return sp.Matrix(self._nu.dot(self._A[:self._N,:self._M]))

def _KerImg(mat):
    """
    Bases of the image and null spaces for a given matrix (composed of integers) by Gaussian elimination.
```

```
    """
    M, N = mat.shape
    def _gaussian(ErMat, m, n):
        "Gaussian␣elimination␣on␣the␣m-th␣row␣for␣except␣for␣the␣first␣n's␣columns."
        headrow  = ErMat[N+m,n:]
        minval   = np.min(np.abs(headrow)[headrow!=0])
        k        = (np.arange(N-n)[np.abs(headrow)==minval])[0]
        if k != 0:
            ErMat[:, [n,n+k]] = ErMat[:, [n+k,n]] # swap
        for i in np.arange(0, N):
            if i == n:
                pass
            else:
                if ErMat[N+m,i] != 0:
                    lcm = np.lcm(headrow[0], ErMat[N+m,i])
                    ErMat[:,i] = ((lcm/ErMat[N+m,i]) * ErMat[:,i]).astype(int)
                    ErMat[:,i] = ErMat[:,i] \
                             - ((lcm/headrow[0]) * ErMat[:,n]).astype(int)
        return ErMat.astype(int)
    ermat = np.concatenate([np.eye(N), mat.copy()], axis = 0).astype(int)
    # Computation for a basis of the kernel
    nonzeroidx = []
    for n in range(N):
        mlist = (np.arange(M)[np.any(ermat[N:,n:], axis = 1)])
        if len(mlist) == 0:
            break
        ermat = _gaussian(ermat, mlist[0], n)
        nonzeroidx.append([N+mlist[0], n])
    for idx in nonzeroidx:
        if (ermat[idx[0], idx[1]] < 0):
            ermat[:, idx[1]] = -ermat[:, idx[1]]
    indice = ~np.any(ermat[N:,:], axis = 0)
    return ermat[:N, indice], ermat[N:, ~indice]

def _AugMat(nu, Xname, mask):
    """
    To construct a symbolic augmented matrix A given a stoichiometric matrix.

    [Input]
        nu      (2D array-like)
                the stoichiometric matrix, of which entries are required to be integers.
        Xname   (1D iterable)
                the name of the system variables.
        mask    (2D array-like)
                pecifies the condition in which partial derivatives of r can be omitted.
    Output:
        spA
            the symbolic augmented matrix A.
    """
    col_stand = lambda mat: (mat / np.gcd.reduce(mat, axis = 0))
    C   = col_stand(_KerImg(nu)[0])
    D   = col_stand(_KerImg(nu.T)[0])
    dim = nu.shape[0]+C.shape[1]
    spA = sp.Matrix(np.zeros([dim, dim]).astype(int))
    if np.all(C.shape):
        spA[:C.shape[0],-C.shape[1]:] = sp.Matrix(C.astype(int))
    if np.all(D.shape):
        spA[-D.shape[1]:,:D.shape[0]] = sp.Matrix(D.T.astype(int))
    for i in range(nu.shape[0]):
        for j in range(nu.shape[1]):
            if not mask[i, j]:
                spA[j,i] = sp.symbols('r_{%s%s}'%(j+1, Xname[i]))
    return spA
```

Filename: example.py

```
import numpy as np
from scipy.optimize import minimize, approx_fprime
from scipy.linalg import eigvals
import matplotlib.pyplot as plt
import warnings
warnings.filterwarnings('ignore')

np.random.seed(19410115)

# Reaction scheme:
# R1:   -> A
# R2: A -> B (affected by B)
# R3: B -> A
# R4: A ->
```

```python
# Stoichiometric matrix
stoichiometry = np.array([[1, -1, 1, -1],
                          [0, 1, -1, 0]])

# Kinetic rates function
def reaction_rates(concentrations, rates=[8, 1, 1, 1], feedback_strength=2.5, kinetics_type='type1'):
    threshold = 1
    feedback_type1 = lambda x: 1 / (1 + np.exp(-feedback_strength * (x - threshold)))
    feedback_type2 = lambda x: 1 / (1 + abs(x) ** (-feedback_strength))

    rates_vector = np.ones(4)
    rates_vector[0] = rates[0]  # R1: -> A
    rates_vector[1] = rates[1] * concentrations[0] * feedback_type1(concentrations[1])  # R2: A -> B

    if kinetics_type == 'type2':
        rates_vector[1] = rates[1] * concentrations[0] * feedback_type2(concentrations[1])

    rates_vector[2] = rates[2] * concentrations[1]  # R3: B -> A
    rates_vector[3] = rates[3] * concentrations[0] ** 3  # R4: A ->

    if kinetics_type == 'type3':
        rates_vector[3] = rates[3] * concentrations[0] ** 4

    return rates_vector

# Initial states for generating the diagrams
initial_conditions = np.random.uniform(low=0, high=2.5, size=(2, 40))

# Values for the feedback strength d
d_values = np.linspace(1, 3, 150)

# Store equilibrium solutions vs d for experiments 1, 2, and 3
equilibria_vs_d = {}

for experiment_id in range(1, 4):
    equilibria_vs_d[experiment_id] = []

    for d_index in range(len(d_values)):
        for condition_index in range(initial_conditions.shape[1]):
            # Minimize the flux strength to find local equilibrium
            result = minimize(
                lambda x: 1e2 * np.linalg.norm(stoichiometry @ reaction_rates(x,
                                               feedback_strength=d_values[d_index] + 2*int(experiment_id==3),
                                               kinetics_type=f'type{experiment_id}')) ** 2,
                x0=initial_conditions[:, condition_index]
            ).x

            # Check if the local minimum is close to zero
            if np.linalg.norm(stoichiometry @ reaction_rates(result,
                                                            feedback_strength=d_values[d_index] + 2*int(experiment_id==3),
                                                            kinetics_type=f'type{experiment_id}')) > 1e-5:
                # Filter out non-zero solutions
                continue
            else:
                # Compute the dominant eigenvalue of the Jacobian
                dominant_eigenvalue = max(eigvals(
                    approx_fprime(result,
                                  lambda x: stoichiometry @ reaction_rates(x, \
                                        feedback_strength=d_values[d_index] + 2 * int(experiment_id == 3),
                                        kinetics_type=f'type{experiment_id}'))).real)
                equilibria_vs_d[experiment_id].append(
                    [result[0], result[1], d_values[d_index] + 2*int(experiment_id==3), dominant_eigenvalue]
                )

    equilibria_vs_d[experiment_id] = np.array(equilibria_vs_d[experiment_id])

fig, axes = plt.subplots(1, 3, sharey=True, figsize=(6, 2.5), dpi=200)
panel_labels = ["(a)", "(b)", "(c)"]

# Loop through each experiment (1, 2, 3)
for experiment_id in range(1, 4):
    eq_data = equilibria_vs_d[experiment_id]
    # Bifurcation threshold
    _ = axes[experiment_id - 1].axvline(2 + 2 * int(experiment_id == 3),
                                        lw = 1, color = "brown", alpha = 0.5, linestyle = "--")
    axty = axes[experiment_id - 1].twiny()
    axty.set_xticks([2 + 2 * int(experiment_id == 3)])
    axty.set_xticklabels([r"$\det$"+r"$\mathbf{A}$"+r"$=0$"], size = 8, color = "brown")
    axty.set_xlim(axes[experiment_id - 1].get_xlim())
    # Scatter plot for unstable equilibria where dominant eigenvalue is negative
    _ = axes[experiment_id - 1].scatter(eq_data[:, 2][eq_data[:, 3] < 0],
                                        eq_data[:, 0][eq_data[:, 3] < 0],
                                        s=1, color='blue', label=r"$X_1$")
    # Line plot for stable equilibria where dominant eigenvalue is non-negative
```

```
    _ = axes[experiment_id - 1].plot(eq_data[:, 2][eq_data[:, 3] >= 0],
                                     eq_data[:, 1][eq_data[:, 3] >= 0],
                                     linestyle='--', color='red', lw=0.8, label=r"$X_2$")
    # Scatter plot for unstable equilibria of $X_2$
    _ = axes[experiment_id - 1].scatter(eq_data[:, 2][eq_data[:, 3] < 0],
                                        eq_data[:, 1][eq_data[:, 3] < 0],
                                        s=1, color='red', label=r"$X_2$")
    # X-axis label and limits for each subplot
    _ = axes[experiment_id - 1].set_xlabel(r"$d$")
    _ = axes[experiment_id - 1].set_ylim(-0.1, 2.75)
    # Panel label in the top right corner
    _ = axes[experiment_id - 1].text(1 + 2 * int(experiment_id == 3), 2.5, panel_labels[experiment_id - 1])

# Create a twin axis on the last subplot to add a shared legend
legend_axis = axes[-1].twinx()
_ = legend_axis.plot([], [], color='blue', label=r"$A$")
_ = legend_axis.plot([], [], color='red', label=r"$B$")
_ = legend_axis.set_yticklabels([])
_ = legend_axis.set_yticks([])
_ = legend_axis.legend(loc='upper right', borderpad=0.5, fontsize="8")

fig.tight_layout()
fig.savefig('example.png', dpi = 150)
plt.show()
```

Filename: STNFexp_1.py

```
from symssa import *
from symssa import _KerImg
import matplotlib.pyplot as plt
from scipy.optimize import minimize
from matplotlib import use
from sklearn.cluster import SpectralClustering

use('TkAgg'); del use
plt.rcParams.update({'font.size': 14})

# ---------- [ Network settings ] ------------------------------------------------------------------------------------------ #

# State vector: X = (S1, S1p, S3, S3p, S6, S6p, N, Np)
# Reaction vector: R = (act_S1, dct_S1, act_S3, dct_S3, act_S6, dct_S6, act_N, dct_N)

stoich_matrix = np.array([                              # || Stoichiometric matrix (nu)
    [-1,  1,  0,  0,  0,  0,  0,  0],
    [ 1, -1,  0,  0,  0,  0,  0,  0],
    [ 0,  0, -1,  1,  0,  0,  0,  0],
    [ 0,  0,  1, -1,  0,  0,  0,  0],
    [ 0,  0,  0,  0, -1,  1,  0,  0],
    [ 0,  0,  0,  0,  1, -1,  0,  0],
    [ 0,  0,  0,  0,  0,  0, -1,  1],
    [ 0,  0,  0,  0,  0,  0,  1, -1]
], dtype=np.int64)

reg_matrix = np.array([                                 # || Regulatory matrix (beta)
    [0, 0, 0, 0.5, 0, 0.5, 0, 0],                              # activation of STAT1 regulated by STAT3+ and STAT6+
    [0, 0, 0, 0,   0, 0,   0, 0],
    # [0, 0, 0, 0,   0, 0,   0, 0],                              # SOCS3 deletion
    [0, 0.5, 0, 0.05, 0, 0, 0, 0.45],                          # STAT3 activation by STAT1+ and NFkB+
    [0, 0, 0, 0,    0, 0, 0, 0],
    [0, 1,  0, 0,    0, 0, 0, 0],                              # STAT6 activation by STAT1+
    [0, 0,  0, 0,    0, 0, 0, 0],
    # [0, 0,  0, 0.45, 0, 0, 0, 0.1],                            # KLF4 deletion
    [0, 0,  0, 0.45, 0, 0.45, 0, 0.1],                         # NFkB activation by STAT3+ and STAT6+
    [0, 0,  0, 0,    0, 0, 0, 0]
]).T * 0.9 # scaling factor

alpha_params = np.array([[1, 1, 1, 1.5]]) * 3          # || Activation parameters (alpha) for STATs and NFkBs
reaction_params = np.array([[1, 1, 1, 1]])              # || Reaction parameters (q)
decay_rate = np.array([[1, 1, 1, 1]]) * 0.15            # || Decay rate (d)

# Sigmoid function for regulatory effects
sigmoid = lambda x, threshold=5, steepness=2: 1 / (1 + np.exp(-steepness * (x - threshold)))

# ---------- [ Symbolic Computations ] ------------------------------------------------------------------------------------- #

# Sigmoid function for symbolic computation
symbolic_sigmoid = lambda x, threshold=5, steepness=1.5: 1 / (1 + sp.exp(-steepness * (x - threshold)))

# Symbolic variables for different species (S1, S1p, S3, S3p, S6, S6p, N, Np)
Xsym = sp.symbols("S_1 S_1^p S_3 S_3^p S_6 S_6^p N N^p")
```

```python
# Reaction rates (R)
reaction_rates = np.zeros((stoich_matrix.shape[1], 1), dtype=object)

# Squared power of the symbolic variables (S1, S3, S6)
power_S = sp.Matrix(sp.Matrix(Xsym).applyfunc(lambda e: e ** 2)[:6:2])

# Concatenating squared symbolic terms with NFkB-related term (N)
power_X = sp.Matrix([power_S, Xsym[6]])

# Setting the activation and decay reactions for odd/even indices
reaction_rates[::2, :] = np.diag(alpha_params.flatten()) @ \
                            (reaction_params.T + power_X).applyfunc(lambda x: 1 / x).applyfunc(lambda x: 1 - x)
reaction_rates[1::2, :] = np.diag(decay_rate.flatten()) @ sp.Matrix(Xsym[1::2])

# Regulatory effects for each species
regulation_matrix = (reg_matrix.T @ sp.Matrix(Xsym).applyfunc(symbolic_sigmoid)).applyfunc(lambda x: 1 - x)

# Element-wise multiplication to compute final reaction rates
R_sym = sp.matrices.dense.matrix_multiply_elementwise(sp.Matrix(reaction_rates), regulation_matrix)

# ---------- [ Jacobian Calculations ] ---------------------------------------------------------------------------------------- #

# Compute the reduced Jacobian J_g
V = sp.Matrix(_KerImg(stoich_matrix)[1])
V_dagger = (V.T @ V).inv() @ V.T
J_f = stoich_matrix @ R_sym.jacobian(Xsym)
J_g = V_dagger @ J_f @ V

A_matrix = CRN(stoich_matrix, kineticmask=~((stoich_matrix < 0) + (reg_matrix > 1e-5)), varinames=Xsym).A
L = np.asarray(CRN(stoich_matrix.astype(np.int64)).A).copy()
L[:stoich_matrix.shape[1], :stoich_matrix.shape[0]] = stoich_matrix.T
D_transpose = sp.Matrix(L[stoich_matrix.shape[1]:, :stoich_matrix.shape[0]])
Kn_matrix = sp.Matrix(L[:stoich_matrix.shape[1], stoich_matrix.shape[0]:])

# Compute the determinant of matrices
L_matrix = sp.Matrix(L).T
det_L = int(sp.Matrix(L_matrix).det())
det_DD = int((D_transpose @ D_transpose.T).det())
det_Kn = int((Kn_matrix.T @ Kn_matrix).det())

# Print determinant results
print("The determinants of Lambda, (D^T D), (Kn^T Kn) are {:d}, {:d}, and {:d}, respectively.".format(det_L, det_DD, det_Kn))

# Compute the coefficient (coef) using the determinants
coef = det_DD * det_Kn / det_L

A_matrix = CRN(stoich_matrix, kineticmask=~((stoich_matrix < 0) + (reg_matrix > 1e-5)), varinames=Xsym).A

# ---------- [ Matrix Reordering ] ------------------------------------------------------------------------------------------- #
# Pr = np.eye(A_matrix.shape[0], dtype=int)
# Pc = np.eye(A_matrix.shape[0], dtype=int)
# Pr[[0, 1, 2, 3, 4, 5, 6, 7, 8, 9, 10, 11], :] = Pr[[6, 7, 11, 4, 5, 9, 0, 1, 8, 2, 3, 10], :]
# Pc[[6, 7, 11, 4, 5, 9, 0, 1, 8, 2, 3, 10], :] = Pc[[0, 1, 2, 3, 4, 5, 6, 7, 8, 9, 10, 11], :]
##  (Pr @ A_matrix @ Pc) is what we used for the consideration of network modifications.

# ---------- [ Numerical Computations ] -------------------------------------------------------------------------------------- #

def compute_Jg_num(state_vector):
    '''Function to compute the numeric reduced Jacobian J_g for a given state vector'''
    Jg_numeric = J_g.copy()
    for i in range(len(Xsym)):
        Jg_numeric = Jg_numeric.subs(Xsym[i], state_vector[i])
    return np.array(Jg_numeric, dtype=float)

def compute_Jf_num(state_vector):
    '''Function to compute the numeric full Jacobian J_f for a given state vector'''
    Jf_numeric = J_f.copy()
    for i in range(len(Xsym)):
        Jf_numeric = Jf_numeric.subs(Xsym[i], state_vector[i])
    return np.array(Jf_numeric, dtype=float)

def compute_R_num(state_matrix):
    '''Function to compute the numeric reaction rates (R) for a given state matrix (Xs)'''
    reaction_matrix = np.full((stoich_matrix.shape[1], state_matrix.shape[1]), np.nan)
    stat_power = state_matrix[0::2, :] ** np.array([[2, 2, 2, 1]]).T
    reaction_matrix[::2, :] = alpha_params.T * (stat_power / (stat_power + reaction_params.T))
    reaction_matrix[1::2, :] = decay_rate.T * state_matrix[1::2]
    regulation_effects = 1 - (reg_matrix.T @ sigmoid(state_matrix))
    reaction_matrix = reaction_matrix * regulation_effects
    return reaction_matrix

def find_zero_nu_R(coupling_q=[10, 10, 10, 10], threshold=1e-3):
    '''Function to find the steady state of the system (when net flow is zero)'''
    coupling_q = np.array(coupling_q)
```

```python
    def flow(theta):
        system_state = np.zeros((8, 1), dtype=float)
        ratio = np.cos(theta) ** 2
        system_state[0::2, 0] = coupling_q * ratio
        system_state[1::2, 0] = coupling_q * (1 - ratio)
        return np.linalg.norm(stoich_matrix @ compute_R_num(system_state)) ** 2
    theta_initial = np.random.uniform(0, 0.5 * np.pi, (4,))
    result = minimize(flow, theta_initial)
    system_state = np.zeros((8, 1), dtype=float)
    ratio = np.cos(result.x) ** 2
    system_state[0::2, 0] = coupling_q * ratio
    system_state[1::2, 0] = coupling_q * (1 - ratio)
    # Check if the computed system is valid (within the threshold and non-negative)
    if np.linalg.norm(stoich_matrix @ compute_R_num(system_state)) >= threshold or np.any(system_state < 0):
        system_state = system_state * np.nan  # Invalid result, set to NaN
    return system_state


# ---------- [ Main Execution Block ] ------------------------------------------------------------------------------------- #

if __name__ == '__main__':
    # Set random seed for reproducibility
    np.random.seed(2000)

    # Define the number of trials and steps for simulations
    num_trials = 40
    num_steps = 35

    # Generate random ratios for the initial concentrations
    ratio = np.random.uniform(0.025, 0.975, (8, num_trials * num_steps))
    ratio[1::2] = 1 - ratio[0::2]  # Ensure paired ratios sum to 1

    # Define a range for eta (parameter standing for STAT1 gene expression)
    eta_range = np.linspace(6, 8.5, num_steps)

    # Initialize the state matrix with repeated eta values
    init_state = np.repeat(np.repeat(eta_range.reshape(1, -1), 8.5, axis=0), num_trials, axis=1)
    init_state[2:, :] = 8.5  # Set values for STAT3, STAT6, and NFkB
    init_state = init_state * ratio  # Apply the random ratios

    # ---------- [ Finding Equilibria (Zero Points) ] ------------------------------------------------------------------------ #

    # Initialize a matrix to store equilibrium points (zeros)
    zero_state = np.full((stoich_matrix.shape[0], num_trials * num_steps), np.nan)
    zero_state[0, :] = np.repeat(eta_range, int(zero_state.shape[1] / eta_range.shape[0]))
    zero_state[[2, 4, 6], :] = 8.5  # Set initial values for STAT3, STAT6, NFkB

    # Compute the zero points for each trial and step
    for i in range(zero_state.shape[1]):
        zero_state[:, [i]] = find_zero_nu_R(coupling_q=zero_state[[0, 2, 4, 6], i])

    # Filter out distinct and non-NaN equilibrium states
    zero_state = np.unique(zero_state.round(6), axis=1)  # Remove duplicates
    zero_state = zero_state[:, ~np.any(np.isnan(zero_state), axis=0)]  # Remove columns with NaN
    zero_state = zero_state[:, np.argsort(zero_state[4, :])]  # Sort by STAT6 values

    # Copy zero_state to a new variable for further processing
    current_state = zero_state.copy()

    # ---------- [ Euler Method for Steady States ] -------------------------------------------------------------------------- #

    # Use Euler's method to iterate towards steady states
    for t in range(5000):
        current_state += 0.02 * (stoich_matrix @ compute_R_num(current_state))

    # Determine which states have converged to steady states
    convergence_labels = (np.linalg.norm(current_state - zero_state, axis=0) < 1e-3)
    unstable_states = zero_state[:, ~convergence_labels]
    stable_states = zero_state[:, convergence_labels]

    # Filter out distinct unstable and stable states
    unstable_states = np.unique(unstable_states.round(6), axis=1)
    stable_states = np.unique(stable_states.round(6), axis=1)

    # ---------- [ Clustering of Steady States ] ----------------------------------------------------------------------------- #

    # Perform spectral clustering on the stable states
    clustering = SpectralClustering(n_clusters=2, assign_labels='discretize', random_state=0).fit(stable_states.T)
    # Re-determine the clusterinbg label according to STAT6 activation (it makes the previous step redundant though)
    clustering.labels_ = (stable_states[5,:] < 5.).astype(int)

    # ---------- [ Plotting the Results ] ------------------------------------------------------------------------------------ #

    # Set up the figure and axes for plotting
```

```python
fig, ax = plt.subplots(4, 2, sharex=True, sharey=True, figsize=(7, 8))
variable_names = [r"STAT1", r"STAT3", r"STAT6", r"NF$\kappa$B"]

# Marker settings for different data points
marker_unstable = {"color": 'k', 's': 1, "marker": ",", "alpha": 0.5}
marker_stable_cluster0 = {"color": 'b', 's': 25, "marker": 'x', "alpha": 0.5}
marker_stable_cluster1 = {"facecolors": 'none', "edgecolors": 'r', 's': 25, "marker": 'o', "alpha": 0.5}

# Bifurcation threshold (manually defined)
bifurcation_threshold = 6.58

# Loop over each variable to plot
for i in range(4):
    # Reorder indices for plotting
    j = [2, 3, 1, 0][i]
    # Sum of STAT1 and STAT1+
    eta1_values_unstable = (np.array([[1, 1, 0, 0, 0, 0, 0, 0]]) @ unstable_states).flatten()
    eta1_values_stable = (np.array([[1, 1, 0, 0, 0, 0, 0, 0]]) @ stable_states).flatten()
    # Plot unstable states
    ax[j, 1].scatter(eta1_values_unstable, unstable_states[i * 2, :], **marker_unstable)
    ax[j, 0].scatter(eta1_values_unstable, unstable_states[i * 2 + 1, :], **marker_unstable)
    # Plot stable states (cluster 0)
    ax[j, 1].scatter(eta1_values_stable[clustering.labels_ == 0],
                     stable_states[i * 2, clustering.labels_ == 0],
                     **marker_stable_cluster0)
    ax[j, 0].scatter(eta1_values_stable[clustering.labels_ == 0],
                     stable_states[i * 2 + 1, clustering.labels_ == 0],
                     **marker_stable_cluster0)
    # Plot stable states (cluster 1)
    ax[j, 1].scatter(eta1_values_stable[clustering.labels_ == 1],
                     stable_states[i * 2, clustering.labels_ == 1],
                     **marker_stable_cluster1)
    ax[j, 0].scatter(eta1_values_stable[clustering.labels_ == 1],
                     stable_states[i * 2 + 1, clustering.labels_ == 1],
                     **marker_stable_cluster1)
    # Set axis labels and vertical lines for bifurcation
    ax[j, 1].set_ylabel(variable_names[i])
    ax[j, 0].set_ylabel(variable_names[i] + r"$^+$")
    ax[j, 1].axvline(x=bifurcation_threshold, linestyle='--', lw=1.5, alpha=0.5, color='brown')
    ax[j, 0].axvline(x=bifurcation_threshold, linestyle='--', lw=1.5, alpha=0.5, color='brown')
    # label the M1 and M2 branches on the STAT1 panel
    branchticks = [ stable_states[ i*2, clustering.labels_ == 1][np.argmax(eta1_values_stable[clustering.labels_ == 1])],
                   stable_states[ i*2, clustering.labels_ == 0][np.argmax(eta1_values_stable[clustering.labels_ == 0])] ]
    ax[j, 1].text(8.25, branchticks[0]+0.5, 'M1', color = 'r', size = 12)
    ax[j, 1].text(8.25, branchticks[1]+0.5, 'M2', color = 'b', size = 12)
    branchticks = [ stable_states[ i*2+1, clustering.labels_ == 1][np.argmax(eta1_values_stable[clustering.labels_ == 1])],
                   stable_states[ i*2+1, clustering.labels_ == 0][np.argmax(eta1_values_stable[clustering.labels_ == 0])] ]
    ax[j, 0].text(8.25, branchticks[0]+0.5, 'M1', color = 'r', size = 12)
    ax[j, 0].text(8.25, branchticks[1]+0.5, 'M2', color = 'b', size = 12)
for axes in ax[0, :]:
    xax = axes.twiny()
    xax.set_xlim(ax[0, 0].get_xlim())
    xax.set_xticks([bifurcation_threshold])
    xax.set_xticklabels([r"$\det$" + r"$\mathbf{A}$" + r"$=0$"], size = 12, color = 'brown')

# Set common x-labels for the bottom plots
ax[-1, 1].set_xlabel(r"$\eta_1=$" + r"STAT1" + r"$+$" + r"STAT1" + r"$^+$")
ax[-1, 0].set_xlabel(r"$\eta_1=$" + r"STAT1" + r"$+$" + r"STAT1" + r"$^+$")
ax[-1, 1].set_ylim(0, 10)

# Adjust layout and save the figure
fig.tight_layout()
fig.savefig("BD_ori.png")

# ---------- [ Computing Determinants ] ------------------------------------------------------------------------------- #

# Initialize arrays to store determinants
det_Jg_unstable = np.zeros((unstable_states.shape[1],))
det_Jg_stable = np.zeros((stable_states.shape[1],))
det_Jf_all = np.zeros((zero_state.shape[1],))

# J_g for unstable states
for i in range(unstable_states.shape[1]):
    det_Jg_unstable[i] = np.linalg.det(compute_Jg_num(unstable_states[:, i]))

# J_g for stable states
for i in range(stable_states.shape[1]):
    det_Jg_stable[i] = np.linalg.det(compute_Jg_num(stable_states[:, i]))

# J_f for all
for i in range(zero_state.shape[1]):
    det_Jf_all[i] = np.linalg.det(compute_Jf_num(zero_state[:, i]))

# ---------- [ Plotting Determinants ] ------------------------------------------------------------------------------- #
```

```
    fig, ax = plt.subplots(1, 1, figsize=(4.5, 7))

    # Sum of STAT1 and STAT1+ for plotting
    eta1_values_unstable = (np.array([[1, 1, 0, 0, 0, 0, 0, 0]]) @ unstable_states).flatten()
    eta1_values_stable = (np.array([[1, 1, 0, 0, 0, 0, 0, 0]]) @ stable_states).flatten()
    eta1_values_all = (np.array([[1, 1, 0, 0, 0, 0, 0, 0]]) @ zero_state).flatten()

    # Plot determinants for unstable states
    ax.scatter(eta1_values_unstable, coef * det_Jg_unstable, label=r'$\det{A}$␣(unstable)', **marker_unstable)

    # Plot determinants for stable states (cluster 0)
    ax.scatter(eta1_values_stable[clustering.labels_ == 0],
               coef * det_Jg_stable[clustering.labels_ == 0],
               label=r'$\det{A}$␣(M2)',
               **marker_stable_cluster0)

    # Plot determinants for stable states (cluster 1)
    ax.scatter(eta1_values_stable[clustering.labels_ == 1],
               coef * det_Jg_stable[clustering.labels_ == 1],
               label=r'$\det{A}$␣(M1)',
               **marker_stable_cluster1)

    # Plot determinants of the full Jacobian for all zero states
    ax.scatter(eta1_values_all, det_Jf_all, label=r'$\det{J_f}$␣(all)',
               facecolors='none', edgecolors='g', marker='^', s=25)

    plt.legend(loc='lower␣right')

    # Customize plot appearance
    ax.set_ylim(-0.3, 0.3)
    ax.set_xticks([6, 7, 8])
    xax = ax.twiny()
    xax.set_xlim(ax.get_xlim())
    xax.set_xticks([bifurcation_threshold])
    xax.set_xticklabels([r"$\det$" + r"$\mathbf{A}$" + r"$=0$"], size = 12, color = 'brown')
    ax.axvline(x=bifurcation_threshold, linestyle='--', lw=1.5, alpha=0.5, color='brown')

    # Add a downward arrow at the bifurcation threshold
    # arrow_position = 1.01
    # ax.scatter([bifurcation_threshold],
    #             [arrow_position * ax.get_ylim()[1] + (1 - arrow_position) * ax.get_ylim()[0]],
    #             marker='v', s=30, color='k', clip_on=False)

    # Add legend and labels
    ax.set_xlabel(r"$\eta_1=$" + r"STAT1" + r"$+$" + r"STAT1" + r"$^+$")

    # Adjust layout and save the figure
    fig.tight_layout()
    fig.savefig("AJ_ori.png")
    plt.show()

# Note: ChatGPT is used but only to adjust variable names and alignment for better the readability of this script.
# ========== [ END OF THE SCRIPT ] ========================================================================================== #
```

Filename: STNFexp_2.py

```
from symssa import *
from symssa import _KerImg
import matplotlib.pyplot as plt
from scipy.optimize import minimize
from matplotlib import use
from sklearn.cluster import SpectralClustering

use('TkAgg'); del use
plt.rcParams.update({'font.size': 14})

# ---------- [ Network settings ] ------------------------------------------------------------------------------------------ #

# State vector: X = (S1, S1p, S3, S3p, S6, S6p, N, Np)
# Reaction vector: R = (act_S1, dct_S1, act_S3, dct_S3, act_S6, dct_S6, act_N, dct_N)

stoich_matrix = np.array([                                # || Stoichiometric matrix (nu)
    [-1,  1,  0,  0,  0,  0,  0,  0],
    [ 1, -1,  0,  0,  0,  0,  0,  0],
    [ 0,  0, -1,  1,  0,  0,  0,  0],
    [ 0,  0,  1, -1,  0,  0,  0,  0],
    [ 0,  0,  0,  0, -1,  1,  0,  0],
    [ 0,  0,  0,  0,  1, -1,  0,  0],
    [ 0,  0,  0,  0,  0,  0, -1,  1],
```

```
    [ 0,  0,  0,  0,  0,  0,  1, -1]
], dtype=np.int64)

reg_matrix = np.array([                                  # || Regulatory matrix (beta)
    [0, 0, 0, 0.5, 0, 0.5, 0, 0],                               # activation of STAT1 regulated by STAT3+ and STAT6+
    [0, 0, 0, 0,   0, 0,   0, 0],
    [0, 0, 0, 0,   0, 0,   0, 0],                               # SOCS3 deletion
    # [0, 0.5, 0, 0.05, 0, 0, 0, 0.45],                           # STAT3 activation by STAT1+ and NFkB+
    [0, 0, 0, 0,    0, 0, 0, 0],
    [0, 1,  0, 0,    0, 0, 0, 0],                               # STAT6 activation by STAT1+
    [0, 0,  0, 0,    0, 0, 0, 0],
    [0, 0,  0, 0.45, 0, 0, 0, 0.1],                             # KLF4 deletion
    # [0, 0,  0, 0.45, 0, 0.45, 0, 0.1],                          # NFkB activation by STAT3+ and STAT6+
    [0, 0,  0, 0,    0, 0, 0, 0]
]).T * 0.9 # scaling factor

alpha_params = np.array([[1, 1, 1, 1.5]]) * 3           # || Activation parameters (alpha) for STATs and NFkBs
reaction_params = np.array([[1, 1, 1, 1]])              # || Reaction parameters (q)
decay_rate = np.array([[1, 1, 1, 1]]) * 0.15            # || Decay rate (d)

# Sigmoid function for regulatory effects
sigmoid = lambda x, threshold=5, steepness=2: 1 / (1 + np.exp(-steepness * (x - threshold)))

# ---------- [ Symbolic Computations ] ------------------------------------------------------------------------------------ #

# Sigmoid function for symbolic computation
symbolic_sigmoid = lambda x, threshold=5, steepness=1.5: 1 / (1 + sp.exp(-steepness * (x - threshold)))

# Symbolic variables for different species (S1, S1p, S3, S3p, S6, S6p, N, Np)
Xsym = sp.symbols("S_1␣S_1^p␣S_3␣S_3^p␣S_6␣S_6^p␣N␣N^p")

# Reaction rates (R)
reaction_rates = np.zeros((stoich_matrix.shape[1], 1), dtype=object)

# Squared power of the symbolic variables (S1, S3, S6)
power_S = sp.Matrix(sp.Matrix(Xsym).applyfunc(lambda e: e ** 2)[:6:2])

# Concatenating squared symbolic terms with NFkB-related term (N)
power_X = sp.Matrix([power_S, Xsym[6]])

# Setting the activation and decay reactions for odd/even indices
reaction_rates[::2, :] = np.diag(alpha_params.flatten()) @ \
                         (reaction_params.T + power_X).applyfunc(lambda x: 1 / x).applyfunc(lambda x: 1 - x)
reaction_rates[1::2, :] = np.diag(decay_rate.flatten()) @ sp.Matrix(Xsym[1::2])

# Regulatory effects for each species
regulation_matrix = (reg_matrix.T @ sp.Matrix(Xsym).applyfunc(symbolic_sigmoid)).applyfunc(lambda x: 1 - x)

# Element-wise multiplication to compute final reaction rates
R_sym = sp.matrices.dense.matrix_multiply_elementwise(sp.Matrix(reaction_rates), regulation_matrix)

# ---------- [ Jacobian Calculations ] ------------------------------------------------------------------------------------- #

# Compute the reduced Jacobian J_g
V = sp.Matrix(_KerImg(stoich_matrix)[1])
V_dagger = (V.T @ V).inv() @ V.T
J_f = stoich_matrix @ R_sym.jacobian(Xsym)
J_g = V_dagger @ J_f @ V

A_matrix = CRN(stoich_matrix, kineticmask=~((stoich_matrix < 0) + (reg_matrix > 1e-5)), varinames=Xsym).A
L = np.asarray(CRN(stoich_matrix.astype(np.int64)).A).copy()
L[:stoich_matrix.shape[1], :stoich_matrix.shape[0]] = stoich_matrix.T
D_transpose = sp.Matrix(L[stoich_matrix.shape[1]:, :stoich_matrix.shape[0]])
Kn_matrix = sp.Matrix(L[:stoich_matrix.shape[1], stoich_matrix.shape[0]:])

# Compute the determinant of matrices
L_matrix = sp.Matrix(L).T
det_L = int(sp.Matrix(L_matrix).det())
det_DD = int((D_transpose @ D_transpose.T).det())
det_Kn = int((Kn_matrix.T @ Kn_matrix).det())

# Print determinant results
print("The␣determinants␣of␣Lambda,␣(D^T␣D),␣(Kn^T␣Kn)␣are␣{:d},␣{:d},␣and␣{:d},␣respectively.".format(det_L, det_DD, det_Kn))

# Compute the coefficient (coef) using the determinants
coef = det_DD * det_Kn / det_L

A_matrix = CRN(stoich_matrix, kineticmask=~((stoich_matrix < 0) + (reg_matrix > 1e-5)), varinames=Xsym).A

# ---------- [ Matrix Reordering ] ----------------------------------------------------------------------------------------- #
# Pr = np.eye(A_matrix.shape[0], dtype=int)
# Pc = np.eye(A_matrix.shape[0], dtype=int)
# Pr[[0, 1, 2, 3, 4, 5, 6, 7, 8, 9, 10, 11], :] = Pr[[6, 7, 11, 4, 5, 9, 0, 1, 8, 2, 3, 10], :]
# Pc[[6, 7, 11, 4, 5, 9, 0, 1, 8, 2, 3, 10], :] = Pc[[0, 1, 2, 3, 4, 5, 6, 7, 8, 9, 10, 11], :]
```

```
##  (Pr @ A_matrix @ Pc) is what we used for the consideration of network modifications.

# ---------- [ Numerical Computations ] ------------------------------------------------------------------------------------ #

def compute_R_num(state_matrix):
    '''Function to compute the numeric reaction rates (R) for a given state matrix (Xs)'''
    reaction_matrix = np.full((stoich_matrix.shape[1], state_matrix.shape[1]), np.nan)
    stat_power = state_matrix[0::2, :] ** np.array([[2, 2, 2, 1]]).T
    reaction_matrix[::2, :] = alpha_params.T * (stat_power / (stat_power + reaction_params.T))
    reaction_matrix[1::2, :] = decay_rate.T * state_matrix[1::2]
    regulation_effects = 1 - (reg_matrix.T @ sigmoid(state_matrix))
    reaction_matrix = reaction_matrix * regulation_effects
    return reaction_matrix

def find_zero_nu_R(coupling_q=[10, 10, 10, 10], threshold=1e-3):
    '''Function to find the steady state of the system (when net flow is zero)'''
    coupling_q = np.array(coupling_q)
    def flow(theta):
        system_state = np.zeros((8, 1), dtype=float)
        ratio = np.cos(theta) ** 2
        system_state[0::2, 0] = coupling_q * ratio
        system_state[1::2, 0] = coupling_q * (1 - ratio)
        return np.linalg.norm(stoich_matrix @ compute_R_num(system_state)) ** 2
    theta_initial = np.random.uniform(0, 0.5 * np.pi, (4,))
    result = minimize(flow, theta_initial)
    system_state = np.zeros((8, 1), dtype=float)
    ratio = np.cos(result.x) ** 2
    system_state[0::2, 0] = coupling_q * ratio
    system_state[1::2, 0] = coupling_q * (1 - ratio)
    # Check if the computed system is valid (within the threshold and non-negative)
    if np.linalg.norm(stoich_matrix @ compute_R_num(system_state)) >= threshold or np.any(system_state < 0):
        system_state = system_state * np.nan  # Invalid result, set to NaN
    return system_state


# ---------- [ Main Execution Block ] -------------------------------------------------------------------------------------- #

if __name__ == '__main__':
    # Set random seed for reproducibility
    np.random.seed(2000)

    # Define the number of trials and steps for simulations
    num_trials = 40
    num_steps = 35

    # Generate random ratios for the initial concentrations
    ratio = np.random.uniform(0.025, 0.975, (8, num_trials * num_steps))
    ratio[1::2] = 1 - ratio[0::2]  # Ensure paired ratios sum to 1

    # Define a range for eta (parameter standing for STAT1 gene expression)
    eta_range = np.linspace(6, 8.5, num_steps)

    # Initialize the state matrix with repeated eta values
    init_state = np.repeat(np.repeat(eta_range.reshape(1, -1), 8.5, axis=0), num_trials, axis=1)
    init_state[2:, :] = 8.5  # Set values for STAT3, STAT6, and NFkB
    init_state = init_state * ratio  # Apply the random ratios

    # ---------- [ Finding Equilibria (Zero Points) ] ------------------------------------------------------------------------ #

    # Initialize a matrix to store equilibrium points (zeros)
    zero_state = np.full((stoich_matrix.shape[0], num_trials * num_steps), np.nan)
    zero_state[0, :] = np.repeat(eta_range, int(zero_state.shape[1] / eta_range.shape[0]))
    zero_state[[2, 4, 6], :] = 8.5  # Set initial values for STAT3, STAT6, NFkB

    # Compute the zero points for each trial and step
    for i in range(zero_state.shape[1]):
        zero_state[:, [i]] = find_zero_nu_R(coupling_q=zero_state[[0, 2, 4, 6], i])

    # Filter out distinct and non-NaN equilibrium states
    zero_state = np.unique(zero_state.round(6), axis=1)  # Remove duplicates
    zero_state = zero_state[:, ~np.any(np.isnan(zero_state), axis=0)]  # Remove columns with NaN
    zero_state = zero_state[:, np.argsort(zero_state[4, :])]  # Sort by STAT6 values

    # Copy zero_state to a new variable for further processing
    current_state = zero_state.copy()

    # ---------- [ Euler Method for Steady States ] -------------------------------------------------------------------------- #

    # Use Euler's method to iterate towards steady states
    for t in range(5000):
        current_state += 0.02 * (stoich_matrix @ compute_R_num(current_state))

    # Determine which states have converged to steady states
    convergence_labels = (np.linalg.norm(current_state - zero_state, axis=0) < 1e-3)
```

```
unstable_states = zero_state[:, ~convergence_labels]
stable_states = zero_state[:, convergence_labels]

# Filter out distinct unstable and stable states
unstable_states = np.unique(unstable_states.round(6), axis=1)
stable_states = np.unique(stable_states.round(6), axis=1)

# ---------- [ Clustering of Steady States ] ---------------------------------------------------------------------------- #

# Perform spectral clustering on the stable states
clustering = SpectralClustering(n_clusters=2, assign_labels='discretize', random_state=0).fit(stable_states.T)
# Re-determine the clusterinbg label according to STAT6 activation (it makes the previous step redundant though)
clustering.labels_ = (stable_states[5,:] < 5.).astype(int)

# ---------- [ Plotting the Results ] ------------------------------------------------------------------------------------ #

# Set up the figure and axes for plotting
fig, ax = plt.subplots(4, 2, sharex=True, sharey=True, figsize=(7, 8))
variable_names = [r"STAT1", r"STAT3", r"STAT6", r"NF$\kappa$B"]

# Marker settings for different data points
marker_unstable = {"color": 'k', 's': 1, "marker": ",", "alpha": 0.5}
marker_stable_cluster0 = {"color": 'b', 's': 25, "marker": 'x', "alpha": 0.5}
marker_stable_cluster1 = {"facecolors": 'none', "edgecolors": 'r', 's': 25, "marker": 'o', "alpha": 0.5}

# Bifurcation threshold (manually defined)
bifurcation_threshold = 7.22

# Loop over each variable to plot
for i in range(4):
    # Reorder indices for plotting
    j = [2, 3, 1, 0][i]
    ax[j, 0].set_yticks([0, 5, 10])
    ax[j, 1].set_yticks([0, 5, 10])
    # Sum of STAT1 and STAT1+
    eta1_values_unstable = (np.array([[1, 1, 0, 0, 0, 0, 0, 0]]) @ unstable_states).flatten()
    eta1_values_stable = (np.array([[1, 1, 0, 0, 0, 0, 0, 0]]) @ stable_states).flatten()
    #
    coloring_switch = 0 if (reg_matrix[5, 6] < 1e-3) else -1
    if (j == coloring_switch):
        marker_unstable['color'] = 'k'
        marker_stable_cluster0['color'] = 'r'
    elif (j == 3):
        marker_unstable['color'] = 'b'
        marker_stable_cluster0['color'] = 'b'
        marker_stable_cluster1['edgecolors'] = 'b'
    # Plot unstable states
    ax[j, 1].scatter(eta1_values_unstable, unstable_states[i * 2, :], **marker_unstable)
    ax[j, 0].scatter(eta1_values_unstable, unstable_states[i * 2 + 1, :], **marker_unstable)
    # Plot stable states (cluster 0)
    ax[j, 1].scatter(eta1_values_stable[clustering.labels_ == 0],
                     stable_states[i * 2, clustering.labels_ == 0],
                     **marker_stable_cluster0)
    ax[j, 0].scatter(eta1_values_stable[clustering.labels_ == 0],
                     stable_states[i * 2 + 1, clustering.labels_ == 0],
                     **marker_stable_cluster0)
    # Plot stable states (cluster 1)
    ax[j, 1].scatter(eta1_values_stable[clustering.labels_ == 1],
                     stable_states[i * 2, clustering.labels_ == 1],
                     **marker_stable_cluster1)
    ax[j, 0].scatter(eta1_values_stable[clustering.labels_ == 1],
                     stable_states[i * 2 + 1, clustering.labels_ == 1],
                     **marker_stable_cluster1)
    # Set axis labels and vertical lines for bifurcation
    ax[j, 1].set_ylabel(variable_names[i])
    ax[j, 0].set_ylabel(variable_names[i] + r"$^+$")
    # Reset the color
    marker_unstable['color'] = 'k'
    marker_stable_cluster0['color'] = 'b'
    marker_stable_cluster1['edgecolors'] = 'r'
    # Set axis labels and vertical lines for bifurcation
    ax[j, 1].set_ylabel(variable_names[i])
    ax[j, 0].set_ylabel(variable_names[i] + r"$^+$")
    ax[j, 1].axvline(x=bifurcation_threshold, linestyle='--', lw=1.5, alpha=0.5, color='brown')
    ax[j, 0].axvline(x=bifurcation_threshold, linestyle='--', lw=1.5, alpha=0.5, color='brown')
    # label the M1 and M2 branches on the STAT1 panel
    for t in range(2):
        branchticks = [ stable_states[ i*2+(t==0), clustering.labels_ == 1][ \
                            np.argmax(eta1_values_stable[clustering.labels_ == 1])],
                        stable_states[ i*2+(t==0), clustering.labels_ == 0][ \
                            np.argmax(eta1_values_stable[clustering.labels_ == 0])] ]
        flag_unbif  = (abs(np.diff(branchticks)) < 1e-3)
        if not flag_unbif:
            ax[j, t].text(8.25, branchticks[0] + (2*t - 1) * (-1 + 2*int(j!=0)*int(j!=2)), 'M1',
```

```
                                color = 'r', size = 12, verticalalignment = 'center')
                ax[j, t].text(8.25, branchticks[1]+1, 'M2',
                                color = 'b', size = 12, verticalalignment = 'center')
            else:
                text  = 'M{:d}'.format(1+(j!=0))
                textc = 'r' if (j==0) else 'b'
                ax[j, t].text( 8.25, branchticks[0]+1, text,
                                 verticalalignment = 'center', color = textc, size = 12)
    for axes in ax[0, :]:
        xax = axes.twiny()
        xax.set_xlim(ax[0, 0].get_xlim())
        xax.set_xticks([bifurcation_threshold])
        xax.set_xticklabels([r"$\det$" + r"$\mathbf{A}$" + r"$=0$"], size = 12, color = 'brown')

    # Set common x-labels for the bottom plots
    ax[-1, 1].set_xlabel(r"$\eta_1=$" + r"STAT1" + r"$+$" + r"STAT1" + r"$^+$")
    ax[-1, 0].set_xlabel(r"$\eta_1=$" + r"STAT1" + r"$+$" + r"STAT1" + r"$^+$")
    ax[-1, 1].set_ylim(-0.5, 10)

    # Adjust layout and save the figure
    fig.tight_layout()
    if (reg_matrix[5, 6] == 0):
        fig.savefig("BD_socs3klf4del.png")
    else:
        fig.savefig("BD_socs3del.png")
    plt.show()

# Note: ChatGPT is used but only to adjust variable names and alignment for better the readability of this script.
# ========== [ END OF THE SCRIPT ] ============================================================================== #
```
